# RNA Taste Is Conserved in Dipteran Insects^☆^^☆☆^

**DOI:** 10.1016/j.tjnut.2023.03.010

**Published:** 2023-03-10

**Authors:** Shinsuke Fujii, Ji-Eun Ahn, Christopher Jagge, Vinaya Shetty, Christopher Janes, Avha Mohanty, Michel Slotman, Zach N. Adelman, Hubert Amrein

**Affiliations:** 1 Department of Cell Biology and Genetics, School of Medicine, Texas A&M University, Bryan, TX, United States; 2 Department of Entomology, College of Agriculture and Life Sciences, Texas A&M University, College Station, TX, United States

**Keywords:** gustatory receptors, RNA taste, ribonucleoside taste, insect, Drosophila, blowflies, mosquitoes, growth, larvae, evolution

## Abstract

**Background:**

Ribonucleosides and RNA are an underappreciated nutrient group essential during *Drosophila* larval development and growth. Detection of these nutrients requires at least one of the 6 closely related taste receptors encoded by the *Gr28* genes, one of the most conserved insect taste receptor subfamilies.

**Objectives:**

We investigated whether blow fly larvae and mosquito larvae, which shared the last ancestor with *Drosophila* about 65 and 260 million years ago, respectively, can taste RNA and ribose. We also tested whether the *Gr28* homologous genes of the mosquitoes *Aedes aegypti* and *Anopheles gambiae* can sense these nutrients when expressed in transgenic *Drosophila* larvae.

**Methods:**

Taste preference in blow flies was examined by adapting a 2-choice preference assay that has been well-established for *Drosophila* larvae. For the mosquito *Aedes aegypti*, we developed a new 2-choice preference assay that accommodates the aquatic environment of these insect larvae. Finally, we identified Gr28 homologs in these species and expressed them in *Drosophila melanogaster* to determine their potential function as RNA receptors.

**Results:**

Larvae of the blow fly *Cochliomyia macellaria* and *Lucilia cuprina* are strongly attracted to RNA (0.5 mg/mL) in the 2-choice feeding assays (*P* < 0.05). Similarly, the mosquito *Aedes aegypti* larvae showed a strong preference for RNA (2.5 mg/mL) in an aquatic 2-choice feeding assay. Moreover, when Gr28 homologs of *Aedes* or *Anopheles* mosquitoes are expressed in appetitive taste neurons of *Drosophila melanogaster* larvae lacking their *Gr28* genes, preference for RNA (0.5 mg/mL) and ribose (0.1 M) is rescued (*P* < 0.05).

**Conclusions:**

The appetitive taste for RNA and ribonucleosides in insects emerged about 260 million years ago, the time mosquitoes and fruit flies diverged from their last common ancestor. Like sugar receptors, receptors for RNA have been highly conserved during insect evolution, suggesting that RNA is a critical nutrient for fast-growing insect larvae.

## Introduction

Although the anatomy and distribution of taste sensory systems of vertebrates and arthropods are quite different, molecular-genetic and physiological investigations over the last 20 years have revealed some universal principles. One of these is that both vertebrates and invertebrates evolved multiple taste receptor gene families, each comprising from a few to many (>60) genes. In general, members of a given receptor family are tuned to a group of chemicals representing a specific taste modality, such as sweet taste or bitter taste [1]. In some cases, however, such as the *Gustatory receptors* (*Grs*) *genes* in insects, different proteins of the same family may recognize ligands from several different taste modalities, such as carbohydrates, which elicit sweet taste, or alkaloids and phenols, which elicit bitter taste sensation [[2], [3], [4]]. Another common theme is that species across a wide spectrum of vertebrates and insects can sense chemicals representing the 6 basic human taste modalities of sweet (carbohydrates), umami (amino acids/proteins), oleogustus (fatty acids), sour (carboxylic acids), salty (sodium chloride), and bitter (alkaloids/phenols), reflecting the shared need of animals to be able to detect and discriminate respective nutrients and avoid toxic and harmful compounds. Finally, receptors tuned to distinct chemical categories are generally expressed in different subsets of taste cells or taste neurons, providing a neural framework for taste discrimination [1,3,4], albeit some exceptions to this rule have been reported.

In *D. melanogaster*, the major group of taste receptors is comprised of 68 proteins encoded by the *Gr* genes [[3], [4], [5], [6]]. About 3 quarters of all *Drosophila Gr* genes are poorly conserved among insects, without obvious orthologs in other families. Most of these diverse *Gr* genes are expressed in gustatory receptor neurons that are activated by nonnutritious compounds such as alkaloids that taste bitter to humans and elicit avoidance behavior in flies. Ca^2+^ imaging studies revealed that these bitter taste receptors function as multimeric complexes composed of at least 1 conserved Gr protein (Gr66a, Gr33a) and 2–3 poorly conserved Grs, which are thought to provide ligand specificity [7,8]. Two of these poorly conserved Grs (Gr32a and Gr68a) have also been implicated in pheromone perception [[9], [10], [11]]. The remaining *Gr* genes that have been functionally characterized in some detail fall into 3, more highly conserved *Gr* subfamilies, the homologs for which are found across all insect families and even more distant arthropods. The overall most conserved Gr proteins are Gr21a and Gr63a, which are expressed in olfactory neurons and form a heteromeric carbon dioxide (CO_2_) receptor [12,13]. A second extensively characterized subfamily consists of the 8 sweet taste receptor genes (*Gr5a*, *Gr61a*, and *Gr64a-Gr64f*), 6 of which are also physically linked within a 20-kb region in the genome [[14], [15], [16]]. Finally, a single conserved Gr protein, Gr43a, was shown to function as the sole sugar receptor in larvae [17], and was also found to be expressed in neurosecretory cells in the larval and adult brain where it functions as a sensor for circulating fructose to regulate feeding behavior [18]. Interestingly, *Gr43a* homologs have also been found to be expressed in nonchemosensory organs (brain and gut) in the silk moth *Bombyx mori* and the cotton bollworm *Helicoverpa armigera* [19,20].

We recently reported on a novel taste modality in *D. melanogaster* larvae, the appetitive taste of RNA and ribonucleosides [21]. We showed that attraction to these chemicals is mediated by one of the 6 Gr28 proteins, encoded by a gene cluster within 10 kb on the second chromosome (*Gr28a*, *Gr28b.a*, *Gr28b.b*, *Gr28b.c*, *Gr28b.d*, and *Gr28b.e*). Moreover, we found that the *Gr28* locus is essential for *Drosophila* to survive when they were presented with a complex food environment that required larvae to navigate between food patches with and without ribonucleosides. We postulated that even though ribonucleosides can be synthesized from carbon precursors, they are essential food compounds for larvae because of their highly accelerated growth from the time of hatching to the time of pupation, during which they double their weight almost twice a day.

The *Gr28* genes exhibit the most unusual expression profile of any *Gr* genes, in addition to being expressed in taste neurons [22]. Specifically, several of them are found in numerous other organ systems, including many neurons in the brain and central nerve cord, other sensory neurons, and cells in the gut [21,22]. Given this diverse expression profile, it is not surprising that they have been associated with functions not related to taste. Specifically, at least one of the *Gr28b* genes has been implicated in UV light sensing in larvae [23], whereas *Gr28b.d* mediates thermotaxis and allows flies to avoid warm temperatures [24].

Because the *Gr28* genes are well conserved among arthropods (see below), we investigated in this paper whether chemosensory function in RNA/ribonucleoside perception is a shared appetitive taste modality in other dipteran insects. We present data showing that blow flies and mosquitoes, which have diverged from a common ancestor shared with *Drosophila* about 65 million [25] and 260 million [26] years ago, respectively, can taste RNA and ribose. Moreover, we found that the taste of RNA and ribose can be rescued in *Drosophila Gr28* mutant larvae by expressing either *Aedes aegypti* or *Anopheles gambiae* homologs. Our findings suggest that conserved taste receptors mediate chemosensory preference for RNA, an insect taste modality critical for identifying RNA and ribonucleoside nutrients to support rapid larval growth.

## Methods

### Maintenance of insects

Blow flies *C. macellaria* and *L. cuprina* (gift from Dr. Aaron Tarone) were raised on fresh beef liver at 23 °C on a 12-h light–dark cycle. *A. aegypti* mosquitoes were raised using powdered fish food (TetraMin) during larval development, with adults given ad libitum access to 10% sucrose and weekly access to defibrinated sheep blood (Colorado Serum Company), with all life stages raised at 28 °C on a 14-h light–dark cycle. *Drosophila melanogaster* was raised on standard cornmeal food at 25 °C on a 12-h light–dark cycle.

### Standard cornmeal food

Agar (10.88 g, Drosophila agar type II Genesee; 62-103), 78 g of corn meal (Genesee, 66-101), 165 g of malt extract (Alternative Beverage, MUN-UL), 41.25 g of yeast extract (Genesee, 62-106), and 4.69 g of propionic acid (VWR, TCP0500-500mL) were dissolved in 1.5 L of water, boiled, and cooled to 60 °C before being supplemented with 0.075 g of chloramphenicol (Sigma-Aldrich, C0378) and 2.11 g of tegosept (Sigma-Aldrich, PHR1012).

### Molecular biology

To generate the mosquito RNA receptor constructs, total RNA was extracted from 4th instar larvae of both *Aedes aegypti* and *Anopheles gambiae* using TRIzol Reagent (Invitrogen), and reverse transcribed with Superscript IV reverse transcriptase (Invitrogen). To amplify full-length coding regions of *AaGr19aa* and *AgGr33*, RT-PCR (98 °C for 30 s, 65 °C for 30 s, 72 °C for 1 min for 35 cycles) was performed using primers 1 and 2 (1335 bp) and primers 3 and 4 (1332 bp), respectively. *EcoRI* and *XbaI* (underlined in sequences below) restriction sites were incorporated into primers for directional cloning. PCR products were subcloned into pUAST [27] and confirmed by DNA sequence analysis. A full-length clone for *Gr19ac* was obtained in 2 steps, as none of the initially amplified primary clones with primers spanning the entire coding sequence (primers 5 and 8) corresponded to the sequence deposited at the NCBI. Thus, a second set of clones was amplified from primary clones with correct sequence in the aminoterminal or carboxyterminal half of the coding sequence with primers 5 and 6 and primers 7 and 8, respectively. Equal amounts of PCR product were then annealed and reamplified with primers 5 and 8, and the full-length clone was processed as described above. Transgenic fly lines were generated by a standard procedure with P-element–mediated transformation of *w*^*1118*^ embryos (BestGene Inc). The primers used were as follows: *1*) forward 5′- TAACTGAATTCATGGCAGTCTCTACATGGTTTCGG -3′; *2*) reverse 5′- AAGCGTCTAGATTATGGTGACAACGTTGTATTCGC -3′; *3*) forward 5′- TAACTGAATTCATGCGGAAGTGTTTGCGAACTTTT -3′; *4*) reverse 5′-AAGCGTCTAGATTACCAAGTTTCGTTGCCAAGGAA -3′; *5*) forward 5′- TAACTGAATTCATGGCATCGAGTCATTGGTTCC -3′; *6*) reverse 5′- ATAGTTAACATTTGCACTTTGAAATAATCCTCGAC -3′; *7*) forward 5′- AATCGTTATATGCCGGATGTTATCAACCAAGTGG -3′; and *8*) reverse 5′- AAGCGTCTAGATTATGGTGACAACGTTGTATTCGC -3′.

### Chemicals

Chemicals used for preference assays were agarose (Apex, 20-102), t-RNA (Roche Diagnostics GmbH, 10109509001), ribose (Sigma-Aldrich, R7500), sucrose (Macron, 8360-06), L-serine (Sigma-Aldrich, S4500), L-alanine (Sigma-Aldrich, A7627) and glycine (G-Biosciences, RC-054), threonine (Sigma-Aldrich, T8441), proline (Thermo Scientific, CAT: A10199.14), and phenylalanine (Sigma-Aldrich, A5482).

### Taste preference assays for blow fly and *Drosophila* larvae

**FIGURE 1:**
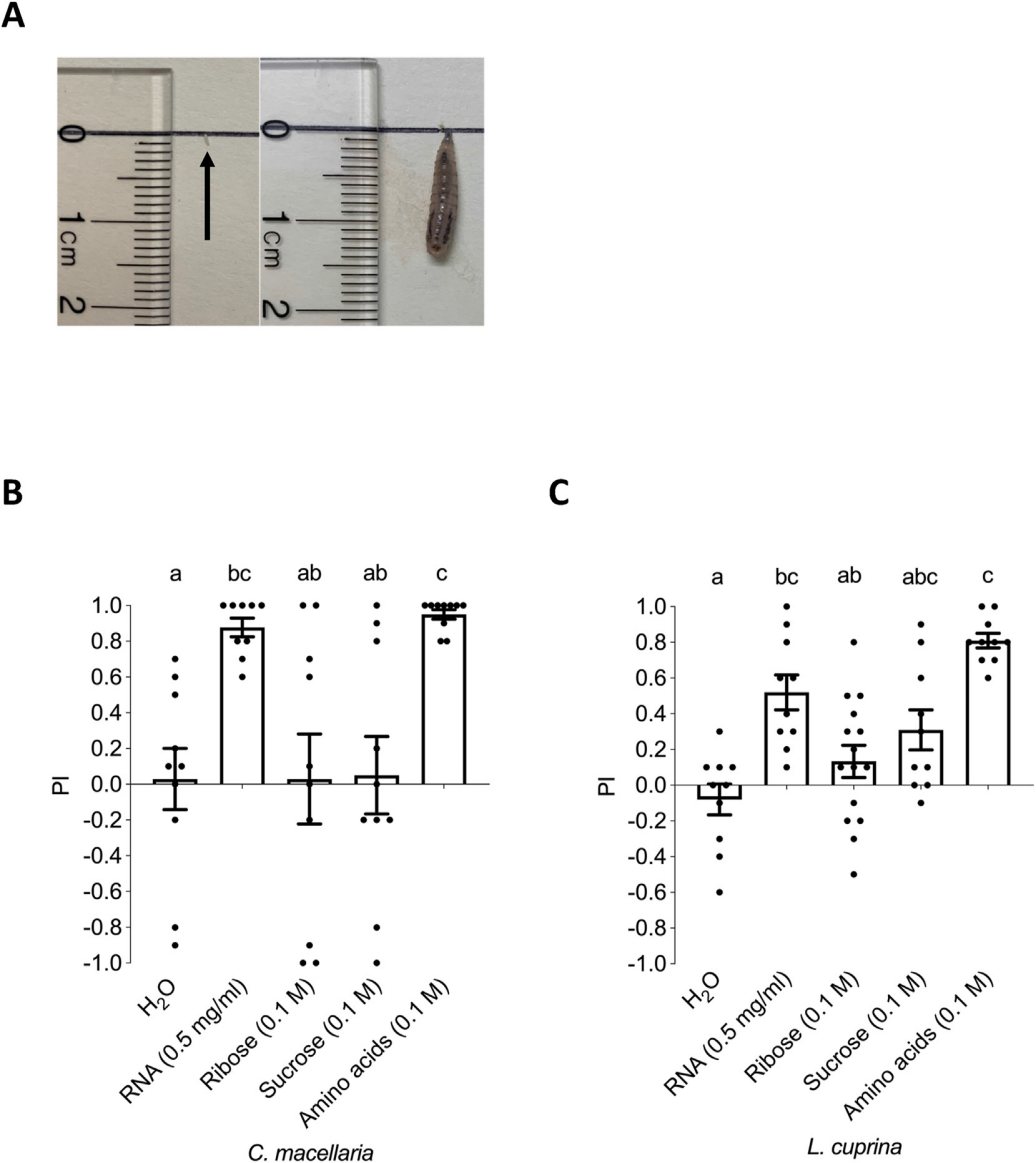
Taste preference and growth of *Calliophoridae* larvae. (A) Growth of blowfly larvae: a larvae hatching from a *C. macellaria* egg, measuring 1 mm in length (black arrow), grows to about 1.5 cm during larval development. (B, C) Taste preference of larvae of the blow flies *C. macellaria* and *L. cuprina*: a positive PI indicates preference for the substrate tested, whereas a negative PI would indicate avoidance. A PI near 0 indicates no preference or avoidance of a substrate (for details, see Methods). Larvae were scored 16 min after they are placed on the midline, demarcating the border of the 2 agarose halves. Both *C. macellaria* (B) and *L. cuprina* (C) larvae are strongly attracted to RNA (0.5 mg/L) and a mixture of 5 amino acids (serine, alanine, glycine, and threonine [100 mM each] and 10 mM phenylalanine). *C. macellaria* showed no behavioral response to ribose or sucrose (0.1 M), whereas some attraction to both these compounds was observed for *L. curprina*. Control assay, against which statistical significance was calculated, were feeding experiments in which both feeding sources were plain agarose (indicated as “H_2_O” lane in Figure 1B, C). Values are expressed as means ± SEM. Different letters indicate statistically significant difference (*P* < 0.05); *n* = 9–10. PI, preference index.

Taste preference assays were performed as described [21]. Briefly, larvae (10 blow fly or 15 *D. melanogaster* larvae) were rinsed in water and placed along the midline of a feeding arena (60 × 15 mm Petri dish) containing freshly prepared 1% agarose on one side and 1% agarose mixed with taste compound on the opposite side. Larvae were scored at 16 min after placing on the midline demarcating the 2 agarose halves. The preference index (PI) was calculated as follows: PI = (number of larvae on agarose with taste compound − number of larvae on agarose without taste compound) / total number of larvae. Statistical significance was determined by comparison to control experiments (lane labeled “H_2_O” in Figure 1B, C), in which both sides of the feeding arena contained pure 1% agarose.

### The preparation of food patches for mosquito 2-choice feeding assay

Agarose food plugs (2 cm diameter × 2.5 mm height, 1% agarose) were stuck on a glass slide (28 × 75 mm). Two slides (one with a substrate containing agarose plug and one with a plain agarose plug) were attached inside a glass tank (10 × 10 × 20 cm), filled with fresh water to a depth of 6.5 cm, using clips to hold the slides in position so that the distance between 2 food plugs was approximately 5 cm.

### Preference assay for mosquito larvae

**FIGURE 2:**
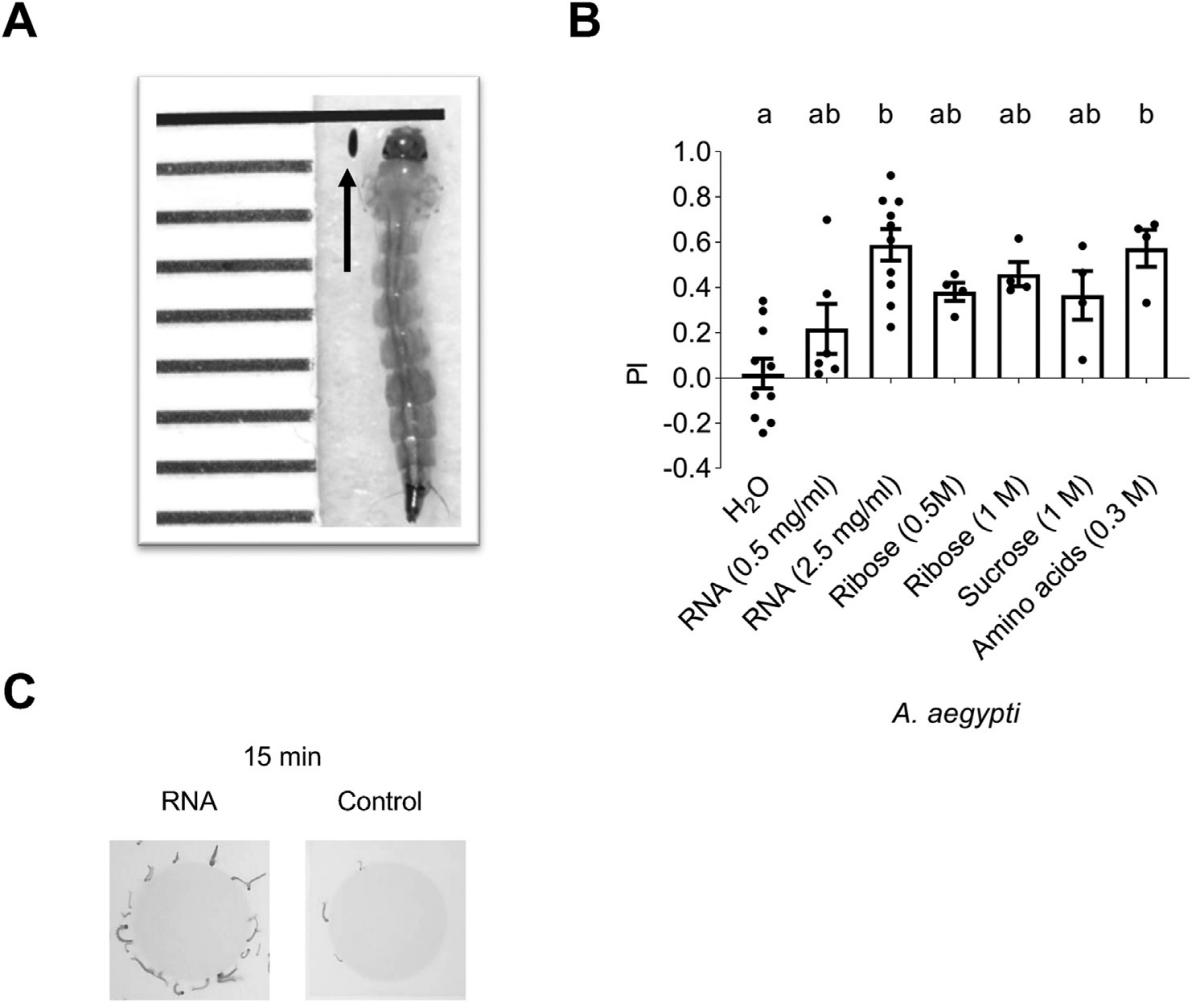
Taste preference and growth of *A. aegypti* larvae. (A) Growth of *A. Aegypti* larvae: size of an *Ae.* aegypti egg (black arrow) and a 4th instar larva shows extreme growth from approximately 0.5 mm to 0.8 cm in about a week. (B) *A. aegypti* larvae attraction to various nutrients: PI for RNA (0.5 and 2.5 mg/mL), ribose (0.5 and 1M), sucrose (1 M) and amino acids (serine, alanine, glycine, threonine, and proline (300 mM each) and 30 mM phenylalanine). PI was calculated cumulatively by recording the location of larvae near plugs every 2 s over 5 min (11–15 min of the recording). Control assay, against which statistical significance was calculated, were feeding experiments in which both feeding sources were plain agarose (indicated as “H_2_O” lane). Values are expressed as mean ± SEM. Different letters indicate statistically significant difference (*P* < 0.05); *n* = 4–10. For more details, see Methods and Supplemental Figure 1. (C) Still images of larvae at the RNA source: typical distribution of larvae near an RNA containing agarose plug (left) and near the plain agarose plug during a feeding experiment. Images shown are representative of an actual experiment and were taken at the end of the 5-min recording (15-min time point). PI, preference index.

Approximately 80 second instar *A. aegypti* larvae (starved for 12 h before the assay) were used in an experiment. After positioning the slides with the food plugs, the tank was visually isolated from the environment using a cover, and video recording was initiated. Larval movement was recorded for 20 min by taking an image every 2 s. The PI was calculated from the accumulated appearance of larvae near a food plug (2.5 cm × 2.5 cm area centered around the plug) using the images continuously for over 5 min (11–15 min) and scored using Image J (version 2.1.0/1.53c). PI for each image was calculated as follows: (the number of larvae near the substrate containing agarose plug A – the number of larvae near plain agarose plug B) / by the total number of larvae near the plugs, averaged for the recorded 5 min. To eliminate any potential positional effects, assays were performed with reverse placement of the plugs each time. Statistical significance was determined by comparison to control experiments, in which both plugs contained pure 1% agarose, indicated as “H_2_O” in the first lane of Figure 2B.

### Sequence comparison of homologs

**FIGURE 3:**
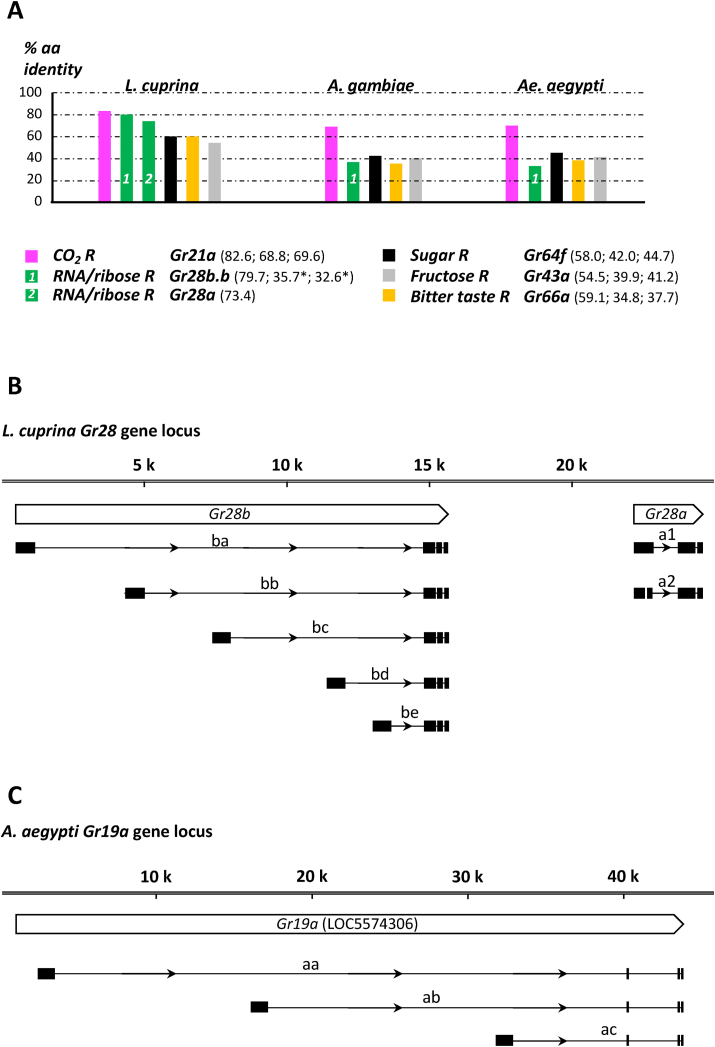
Conservation of well-studied Gr proteins between *Drosophila*, blow flies, and mosquitoes. Evolutionary conservation is presented as the percentage of amino acid identity of the *D. melaogaster* CO_2_ receptor Gr21 (magenta), the Gr28a/Gr28b.b RNA/ribose receptor (green), the sugar receptor Gr64a/Gr64f (black), the bitter receptor Gr66a (orange), and the internal nutrient/larval sugar receptor Gr43a (gray) to their homologs in *L. cuprina*, *A. gambiae*, and *A. aegypti*. The numbers in parenthesis indicate percentage amino acid identity of the *Drosophila* protein to the *L. cuprina*, *A. gambiae*, and *A. aegypti* homologs, respectively. For accession numbers and comparison of all genes within a Gr family, see Supplemental Table 1 and Methods. Gr, gustatory receptor.

Identification of the closest homolog in the 5 categories (CO_2_ receptor, RNA receptor, sugar receptor, bitter receptor, and internal nutrient/larval sugar receptor) between *Drosophila* and *Lucilia*, *Drosophila* and *Aedes*, and *Drosophila* and *Anopheles* (Figure 3) was conducted by first using the deposited amino acid sequence of the *Drosophila* genes in each category (8 sugar receptors, 6 RNA receptors, the CO_2_ receptor Gr21a, the bitter receptor Gr66a, and the internal nutrient/larval sugar receptor Gr43a as a query [Supplemental Table 1]) and searching the NCBI database using protein BLAST. The top match for each of the 5 categories was then used to perform a reverse protein BLAST of the *Drosophila* protein database. The best match for each of the 5 receptor subfamilies was then plotted using the percentage of identical amino acids across at least 90% of the coding sequence. Accession numbers are listed in Supplemental Table 1. Clustal Omega, the EMBL-EBI multiple sequence alignment tool, was used to compare protein sequences, and all parameters shown in Supplemental Table 1 were derived from such analysis.

**FIGURE 4:**
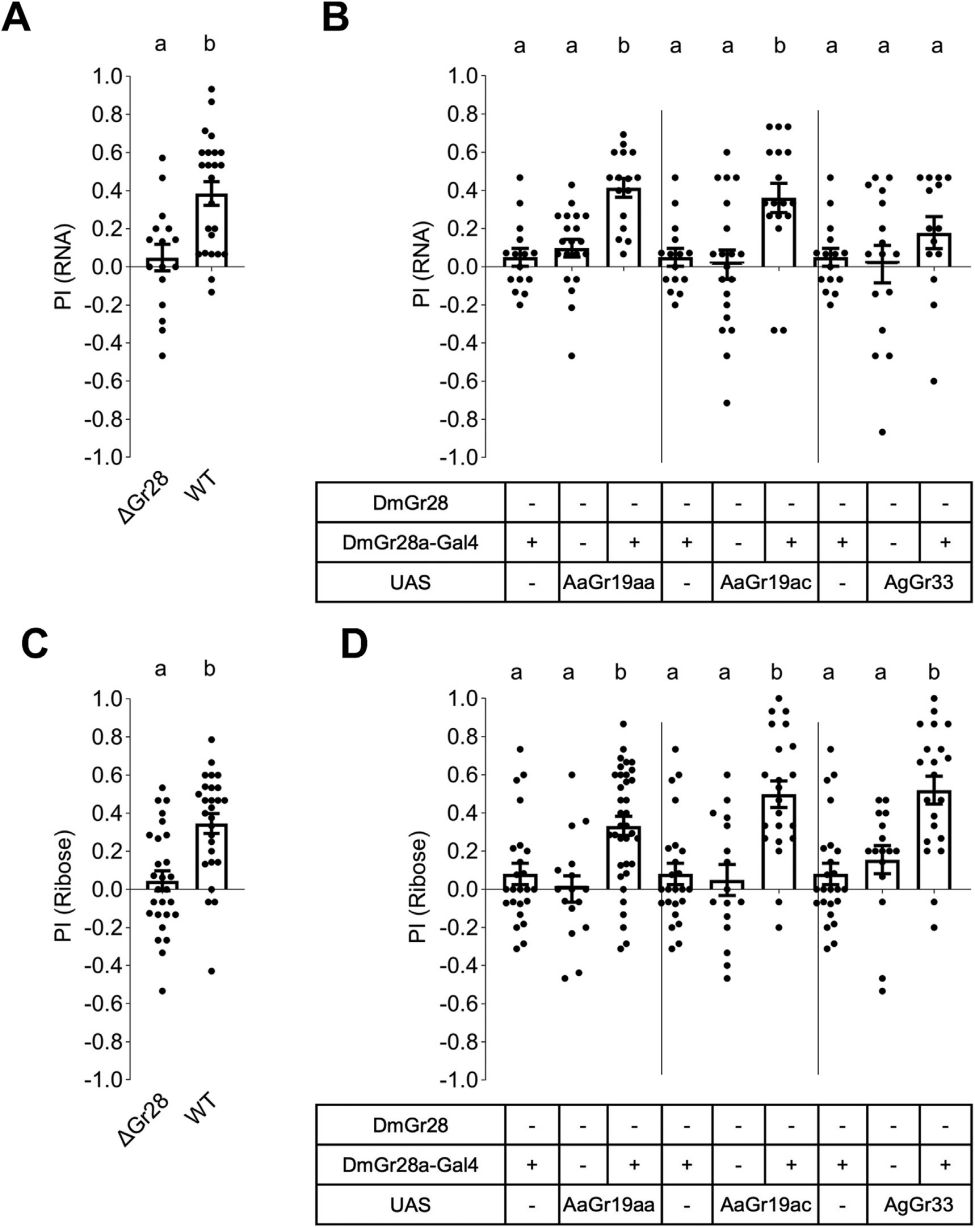
Mosquito Gr28 homologs restore preference to RNA and ribose in *DeltaGr28 D. melanogaster* larvae. Two-choice preference assay to RNA (A, B) and ribose (C, D) of transgenic *Drosophila* lacking the *Gr28* locus, but expressing *AaGr19aa*, *AaGr19ac*, or *AgGr33* under the control of *Gr28a-GAL4*. The location of larvae was scored 16 min after the start of assay. For wild-type larvae (lane 1), control was *DeltaGr28* mutants (lane 1 in A and C). For transgenic larvae expressing a mosquito receptor (lanes 3, 6, and 9), controls were larvae having only the GAL4 driver (lanes 1, 4, and 7) and the respective UAS reporter gene (lanes 2, 5, and 8). Note that the GAL4 driver only control was the same data set for each comparison. Values are expressed as mean ± SEM. Different letters indicate statistically significant difference (*P* < 0.05); *n* = 12–24 (A, B); *n* = 16–36 (C, D). Gr, gustatory receptor.

### Statistical analysis

For each assay, statistical analysis was performed with Prism software suite using the Kruskal–Wallis test for comparing a control to ≥2 experimental data sets (FIGURE 1, FIGURE 2, and Figure 4B, D) and the Mann–Whitney test for comparing a control to a single experimental set (for Figure 4A, C). All values are mean ± SEM. As controls for the experiments shown in FIGURE 1, FIGURE 2, we used plates in which both sides contain plain agarose (H_2_O lane in FIGURE 1, FIGURE 2B). For all experiments, statistical significance was considered if *P* < 0.05.

### Genetics

The genotypes of *D. melanogaster* used in Figure 4 were as follows (number in parenthesis indicates lane): Figure 4A, C: *DeltaGr28*/*DeltaGr28* (1), *w*^*1118*^ (2); Figure 4B, D: *Gr28a-Gal4 DeltaGr28*/*DeltaGr28* (1, 4, 7), *DeltaGr28*/*DeltaGr28 UAS-AaGr19aa/+* (2), *Gr28a-Gal4 DeltaGr28*/*DeltaGr28*; *UAS-AaGr19aa/+* (3), *DeltaGr28*/*DeltaGr28; UAS-AaGr19ac/+* (5), *Gr28a-Gal4 DeltaGr28*/*DeltaGr28; UAS-AaGr19ac/+* (6), *DeltaGr28*/*DeltaGr28; UAS-AaGr33/+* (8), and *Gr28a-Gal4 DeltaGr28*/*DeltaGr28; UAS-AgGr33/+* (9).

## Results

### Larvae of blow flies can sense and are attracted to RNA

We first tested the larvae of 2 different blow fly species, *Cochliomyia macellaria* and *Lucilia cuprina*, for their ability to sense RNA. Both species belong to the family *Calliphoridae* and are known to feed on amino acid/protein-rich diets: females require protein for the production of eggs, which they lay on carrion, providing larvae also with a protein-rich diet. Similar to *D. melanogaster*, *C. macellaria* and *L. cuprina* larvae that hatch from tiny eggs grow rapidly through 3 larval stages and form a puparium within a few days. For example, eggs of *C. macellaria* and *L. cuprina* are only about 1 mm long, but larvae emerging from these eggs grow to about 1.5–2 cm within a week (Figure 1A). We employed the same 2-choice feeding assay used for the *D. melanogaster* larvae [17,21] and first examined larval preference for amino acids. As expected, both *C. macellaria* and *L. cuprina* showed a strong preference for amino acid–containing agarose (Figure 1B, C). Additionally, *L. cuprina* showed some attraction to sucrose and ribose, whereas *C. macellaria* did not. Intriguingly, RNA was as highly desirable as amino acids for both species. Thus, RNAs, like amino acids, are highly desirable nutrients for larvae of the blow fly species *C. macellaria* and *L. cuprina*.

### *Aedes aegypti* is attracted to diverse food chemicals, including ribose and RNA

*Calliphoridae* and *Drosophilidae* diverged from a common ancestor about 65 million years ago [25], and they share similar developmental profiles and anatomy. To examine whether the preference for RNA is conserved in evolutionarily more distant dipteran species, we examined the taste behavior of *A. Aegypti* mosquito larvae, a member of the *Culicidae* family, which diverged from a common ancestor with *Drosophila* about 260 million years ago [25,26]. Consequently, differences in development, anatomy, and behavior are more pronounced, with one of the most striking being the aquatic life of mosquito larvae. However, like blow and fruit fly larvae, mosquito larvae also eat ferociously during the 4 larval stages, growing from about 0.5 mm to close to 1 cm within a week (Figure 2A). Thus, if RNA is indeed a nutrient required for fast-growing insects, we expect mosquitoes to have acquired a taste for RNA as well. Therefore, we developed a 2-choice feeding assay that can accommodate an aquatic habitat (Supplemental Figure 1). We monitored the location of larvae near 2 agarose patches, 1 plain, and the other containing a nutrient substrate, at 2-s intervals for 5 min in a visually controlled environment and calculated the aquatic larvae preference index (see Methods; Figure 2B). Using sugars and amino acid mixtures, we established the suitability of the assay and found that mosquito larvae are attracted to these nutrients in a dosage-dependent manner (Figure 2B, Supplemental Figure 1). We then examined the attraction to RNA and ribose and found that they too were attractive for *A. aegypti* larvae (Figure 2B, C). Thus, the taste of RNA is a broadly acquired larval taste modality of diverse insect families, including *Calliphoridae*, *Drosophilidae*, and *Culicidae.*

### RNA receptors are conserved in blow flies and mosquitoes

In *D. melanogaster*, RNA taste is mediated through members of the Gr28 protein subfamily, encoded by 6 tightly clustered genes (*Gr28a* and *Gr28b.a* to *Gr28b.e*) within 10 kilobases (kb). *Gr28a* is a single gene downstream of the *Gr28b* locus, which is a complex transcription unit characterized by 5 distinct promotors and unique first exons that are spliced to common 2nd and 3rd exons, resulting in 5 closely related receptors with conserved but distinct amino terminal domains and an identical carboxyterminal domain [21,22]. The role of *Gr28* in RNA taste was initially established using a deletion of the entire locus (*DeltaGr28*), which leads to a loss of ribose/RNA sensing in *Drosophila* larvae, a phenotype that can be rescued by expressing single *Gr28* genes, such as *Gr28a*, *Gr28b.a*, or *Gr28b.c* in *Gr28a* neurons [21]. These observations indicate that the RNA sensing function of Gr28 proteins is at least partially redundant and mediated by neurons expressing one of the 6 genes, *Gr28a*.

While the overall structure of Gr proteins is conserved—they all have 7 trans membrane spanning domains and are about 400–500 amino acids in size—most of the *Drosophila* Grs have diverged enough during evolution that it is not possible to identify clear homologs in more distant dipteran species, such as blow flies or mosquitoes. The exceptions are about a dozen conserved *Gr* genes, the homologs for which are found in most insects and many other arthropods. These *Gr* genes encode receptors for universally important ligands and can be divided into 4 functionally distinct groups in *Drosophila*: 8 sugar receptors (encoded by *Gr5a*, *Gr61a* and *Gr64a-f*), an internal nutrient sensor/larval sugar receptor (*Gr43a*), 2 core subunits of multimeric bitter taste receptors (*Gr66a*, *Gr33a*) and the carbon dioxide receptors (*Gr21a* and *Gr*6*3a*). We wondered how well the Gr28 proteins are conserved when compared with these 4 other groups of conserved Gr proteins and interrogated NCBI sequence databases for putative homologs in *L. cuprina*, *A. aegypti*, *and Anopheles gambiae* using protein BLAST with the 6 *Drosophila* Gr28 amino acid sequences as queries (full sequence of *C. macellaria* is not available yet). We considered genes true homologs if the reverse protein BLAST returned any of the 6 *Drosophila* Gr28 proteins as the highest match. For comparison and as a test of principle, we employed the same search strategy using *Drosophila* genes belonging to the 4 conserved Gr groups (see above). For example, the 2 most conserved *Gr* genes, *Gr21a* and *Gr63a*, for which specific functions have been well-established as heteromeric carbon dioxide receptors not only in *Drosophila* but also in both *Aedes* and *Anopheles* mosquitoes [12,13,28,29], have clear homologs in virtually all insect genomes examined. Indeed, both *Gr21a* and *Gr63a* are very well conserved, with amino acid sequence identity of around 80% between *D. melanogaster* and *L. cuprina*, and of about 70% between *D. melanogaster* and the 2 mosquito species (Figure 3, Supplemental Table 1). Homologous genes were also easily identified for the well-characterized sugar receptor genes (*Gr5a*, *Gr61a*, and *Gr64a-f*), the *Gr43a* gene encoding the internal fructose sensor and larval sugar receptor, and the core bitter taste receptor gene *Gr66a*, with amino acid sequence identity to *L. cuprina* proteins in the range of 55%–60% and to the more distant mosquito proteins at somewhat lower levels (32.5%–45%; Figure 3A, Supplemental Table 1). We note that only 2 complete protein sequences for sugar receptors were annotated in the NCBI database (LcGr64d and LcGr64e), whereas several were annotated as truncated fragments or missed entirely. However, using gene software analysis tools (SnapGene, GenomeScan, etc.), we found that each of the 8 *D. melanogaster* sugar receptors had a homolog in *L. cuprina* (Supplemental Figure 2). When the amino acid sequences of the 6 *Drosophila* Gr28 proteins were queried, homologs were found in all 3 species. Like in the sugar receptors, several not yet annotated putative RNA/ribose receptors in *L. cuprina* were easily identified using gene software analysis tools (Supplemental Figure 2). Surprisingly, sequence identity levels between the RNA receptors of *L. cuprina* and *Drosophila* are in the same range as that of the carbon dioxide receptor (73% for Gr28a and 79% for Gr28b). Remarkably, the overall gene structure was precisely maintained between *D. melanogaster* and *L. cuprina*, with 5 *LcGr28b* genes alternatively transcribed and spliced in the same fashion as the *Drosophila Gr28b* genes, followed by the single *LcGr28a* gene (Figure 3B) [22]. The putative RNA receptors in the mosquito genomes were encoded by 3 genes in *Ae. Aegypti*, *Ag19aa*, *Ag19ab*, *and Aa19ac*, transcribed from distinct promotors and distinct, large first exons that contained most of the coding sequence, which are spliced to shared small exons encoding the C-terminus in each protein (Figure 3C). A single homolog, *AgGr33*, was identified in *A. gambiae*. As expected, the amino acid conservation between the mosquito and *D. melanogaster* genes was lower, with the most conserved pairs being AgGr33*/*Gr28b.b (36%), followed by AaGr19aa/Gr28a and AaGr19ac/Gr28b.b pairs (each 33%), a range comparable to that for the bitter receptors (Figure 3A, Supplemental Table 2). However, the gene organization of the mosquito RNA homologs was not conserved when compared with the fruit fly, suggesting that events leading to the increase in gene number and the alternative splicing structure occurred after the divergence of the mosquito from the fly lineage, but before the separation of *Lucilia* and *Drosophila*.

### *AaGr19aa*, *AaGr19ac*, *and AgGr33* function as RNA and ribose receptors in transgenic *Drosophila*

To test whether the mosquito genes encode ribose/RNA receptors and are the likely cause for *Ae. aegypti’s* ability to sense these compounds (Figure 2), we cloned 2 *Aedes* (NP_001345039.1 and NP_001345038.1) and the single *A. gambiae* homologs (XP_040166293.1) by RT-PCR and expressed them in *Gr28a* neurons of *DeltaGr28* larvae using the GAL4/UAS expression system (Figure 4). *DeltaGr28* is a deletion of the *Gr28* locus and renders *Drosophila* larvae unable to sense RNA or ribose [21] (Figure 4A and 4C). Taste preference for RNA and ribose of the larvae expressing either of the *Aedes* transgenes was rescued significantly when compared to controls (Figure 4B and 4D, left 2 panels; *P* > 0.05) reaching a PI of about 0.4, which is similar to that of wild-type larvae (Figure 4A, C). Similarly, the expression of *AgGr33* in *DeltaGr28* larvae also rescued ribose sensing to the level of wild-type larvae (Figure 4D), while RNA sensing increased only modestly (Figure 4B). In summary, our data provide evidence that the taste of ribose and RNA is conserved across a wide spectrum of dipteran insects and is mediated by conserved taste receptors.

## Discussion

The ability of insects to identify and discriminate between different food chemicals is critical, particularly during larval growth and development. Behavioral analyses of 2 species of *Calliphoridae*, *L. cuprina*, *C. macellaria*, and the *Culicidae A. aegypti*, which shared a common ancestor about 260 million years ago, revealed that they all can sense RNA and amino acids. However, some notable differences in robustness are evident. *C. macellaria* exhibited the most extreme preference behavior, showing no response to sucrose and ribose at concentrations that elicit strong behavioral responses in *D. melanogaster* [17] and mosquito larvae (Figure 2) while being highly attracted to RNA and amino acids (Figure 1). *L. cuprina* attraction to both RNA and amino acids was also strong, albeit slightly less than that of *C. macellaria*, and these larvae showed some appetitive responses to both sucrose and ribose (Figure 1). Remarkably, larvae of more distantly related *Ae. aegypti* were attracted to all these chemicals (Figure 2). Appetitive behavior of *A. aegypti* larvae to RNA is consistent with reports from the pregenomic era, which demonstrated that *Culex pipiens* mosquito larvae show chemotaxis toward ribonucleotides and ribonucleosides [30] and require these compounds in their diet for proper larval growth [31,32].

Based on the ability of the conserved mosquito homologs to restore RNA and ribose taste of *DeltaGr28* mutant *D. melanogaster* larvae (Figure 4), we propose that RNA taste is a discrete taste modality in many dipteran insects and is mediated by a specific set of taste receptors. Since the separation of *Drosophilidae* and *Culicidae* from a common ancestor some 260 million years ago, most taste receptors of the Gr family have diverged so much that homologs cannot be identified based on sequence comparison, except for receptors serving functionally important and common roles. Our analysis shows that the RNA receptors (Gr28, AgGr33, and AaGr19) belong to this group, having maintained primary amino acid sequence similarity comparable to that observed for the Gr66a core subunit of bitter taste receptor complexes (Figures 3). The fact that related *Drosophila* and mosquito receptors sharing “only” 35% sequence identity have maintained the ability to sense RNA is not surprising, considering that several *Drosophila Gr28b* genes, which share about the same level of amino acid sequence identity with *Gr28a* (Supplemental Table 2), can rescue RNA and ribose taste in *DeltaGr28* larvae when expressed in appetitive *Gr28a* neurons [21].

Intriguingly, selective pressure for more stringent sequence conservation of the *Gr28* genes increased later in the dipteran expansion, sometime before *Lucilia* and *Drosophila* separated from a common ancestor about 65 million years ago. Specifically, RNA/ribose receptors remained as similar among these flies as the highly conserved CO_2_ receptors (Figure 3A), the most conserved insect Gr proteins [[33], [34], [35]]. We further note that the *Gr28* gene structure has also been maintained completely between *L. cuprina* and *D. melanogaster*, including the location of the intron that separates the unique first exons from the shared exon 2 (Figure 3B) [22]. However, it seems unlikely that the high conservation of the *Gr28* clade was driven solely or even mainly by the need of these receptors to sense RNA and ribose, given their comparatively low conservation in mosquitoes. Rather, we suggest that these receptors have acquired additional important functions in the *Drosophilidae* and *Calliphoridae* linages, some of which have been identified already in *D. melanogaster* (light sensing, temperature sensing) [23,24]. It is worth noting that *Gr28a* gene expression does not overlap with the expression of the *Gr28b* genes in the larval taste system [21]. In fact, all *Gr28b* expressing neurons also co-express the bitter receptor gene *Gr66a* (Ahn and Amrein, in preparation). Behavioral experiments using larvae in which *Gr28b* neurons were inactivated indicate that they are required for avoidance behavior, suggesting that the *Gr28b* genes have roles in *Drosophila* bitter taste (Ahn and Amrein, in preparation). Thus, it will be interesting to investigate their expression and functions in blow flies and other insects where they have been conserved at such high levels.

Until very recently, virtually nothing was known about the taste or dietary requirement of RNA and ribonucleosides in mammals. This changed with the timely report by Toda et al. [36], who found that the primate glutamate (GLU) sensor T1R1/T1R3 evolved from what appeared to be a receptor tuned strongly to ribonucleotides in nonprimate insectivorous monkeys (Marmoset, Squirrel monkey, etc.). Specifically, they reported a shift in responses of the T1R1/T1R3 receptor of nonprimate monkeys, which respond strongly to inosine monophosphate (IMP), an abundant compound of insects, toward GLU alone or GLU/IMP mixtures in primate T1R1/T1R3 receptors, with glutamate being a key nutrient of leaves. Thus, it will be interesting to examine the ability of other mammals to sense IMP, ribonucleotides, and RNA in more detail.

## Data Availability

Data in this article will be made available upon request. Sequences of newly identifed putative Gustatory receptors in the genomes of Aedes aegypti and Lucila cuprina will be deposited to the NIH Gemone database.
