# Supplementary material for: RNA Taste Is Conserved in Dipteran Insects
Source: J Nutr. 2023 Mar 10;153(5):1636–45. doi: 10.1016/j.tjnut.2023.03.010 (PMC10273160; doi:10.1016/j.tjnut.2023.03.010)
Supplement: Multimedia component 1 [file mmc1.pdf]

Supplementary Figure 1

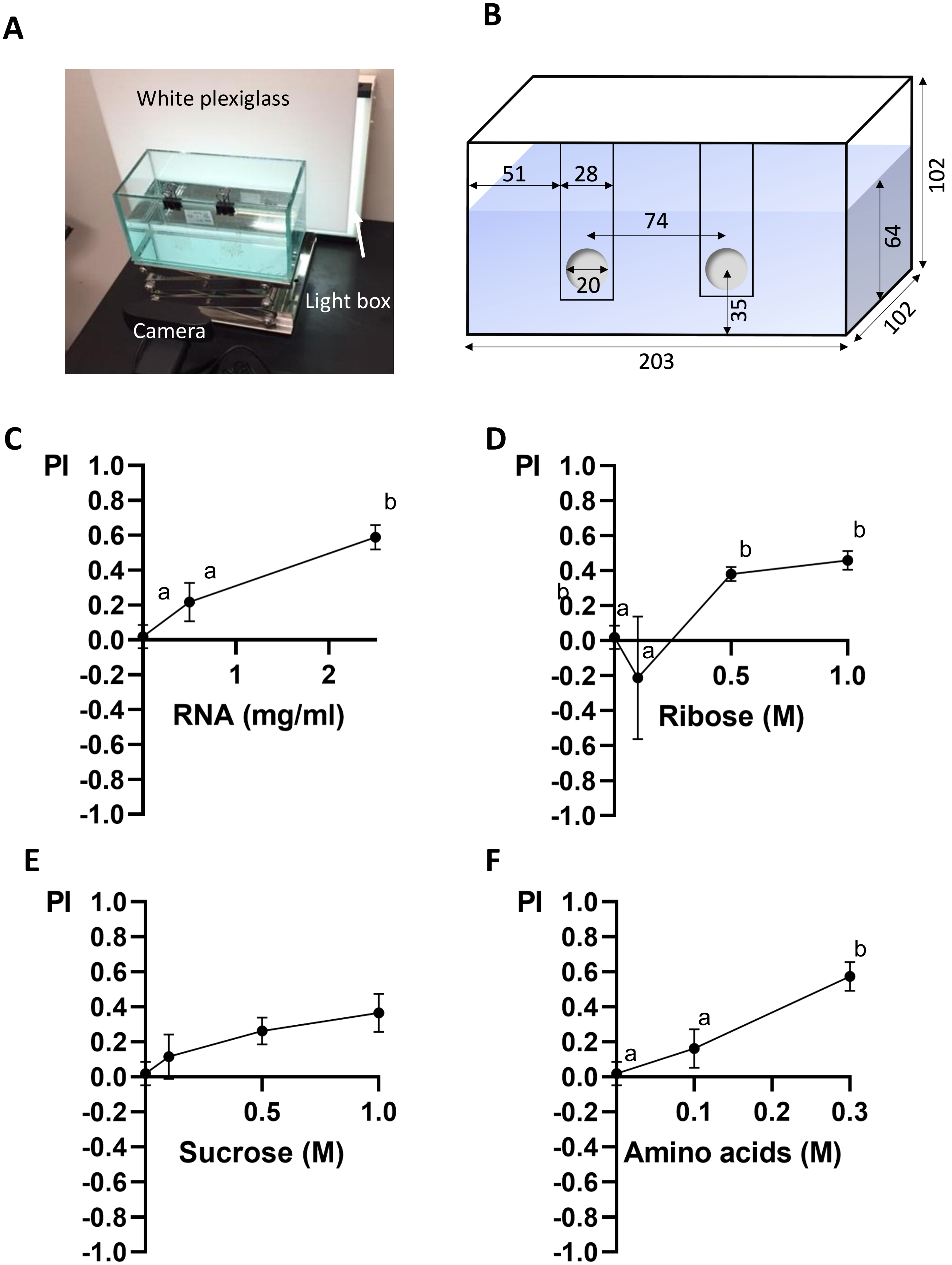

RNA Taste is Conserved in Dipterian Insects: Shinsuke Fujii et al.,

Behavioral taste preference set up for *A. aegypti* larvae. Actual set up (1) and diagram with dimensions (B) of water tank used in the two-choice preference assay. Dimensions are shown in mm; agarose plugs (gray circles) were stuck on microscope slides, which were then positioned inside the tank and kept in place using clamps.
C-F) Dose response profile of larvae shown as preference index (PI) to RNA (0.5 and 2.5 mg/ml, C, ribose (0.1, 0.5 and 1 M, D, sucrose (0.1, 0.5 and 1 M, E) and amino acids (serine, alanine, glycine, threonine, proline 100mM each and 10 mM phenylalanine; and 300mM/30 mM, F). n = 4-10. For details on calculating the PI, see Figure 2 and Methods. Different letters indicate statistically significant difference in preference, $p < 0.05$.

RNA Taste is Conserved in Dipterian Insects: Shinsuke Fujii et al.,

Supplementary Figure 2

L. Cuprina putative RNA/ribose receptor protein and sugar receptor protein sequences. The protein sequences of these genes are currently not/not correctly annotated at NCBI. Below is the predicted amino acid sequence of these genes, using guides (intron exon structure, homology) from the homologous *Drosophila* genes.

RNA/ribose receptors

LcGr28ba

MSDIVSVKSNAWYNRLFNKLFTSDNYYKSMQAMFFLTFIYGITPFCVVSNSYGCKSLKTFYFGYLNAILHICVMA
FCYAYTMYYNESVAGYFLSNNISKLGNKLYVFSGVIGTTVVFVSAVIRTKILQKCFNILLKVDECFKQINYVLDYTLI
LRFTLFVLSAVALFVGTLAVICVYCLKSMNVYPSPCLIVIVVAEFLTISVSISLFCSMTRSVQRRVRLLNTVLKNLCH
QWDTRSIKTITHKQRSLQCLDSFSMYTIVSKNPAEIIQESMEIHQLICEAASTANKYFTYQLLTIISIAFLIIVFDAYY
VLETLLGKSKRESKFKTVEFVTFFSCQMILYLIAIISIVEGSNRAIKKSEKTGGIVHALLNKAKTADVKEKLQQFSMQ
LMHLKINFTAAGLFNIDRTLYFTISGALTTYLIILLQFTSNSPPVTQNGCDPALAVSLNQTET

LcGr28bb

MFSQIVSDKIDRFRKSFISNQVFEALQPLFFLTFLYGLTPFRVIKNKDGESNVQMSFFGFVNIAVYILLYGCCYIISLL
QDETMVGYFFRTKISNVGDTMQICNGLITGAVIYISAVTQRRKLLRVCRILYSLDTNFANIGIKVKYSRIYRYSIVMII
FQTVVIGVYFAGVFRLLKSMKVTPSFSVCVTFFLQHSVLSIAICLFCFMARSFERRLVILNKVLKNLCHQWDTRSIK
TITHKQRSLQCLDSFSMYTIVSKNPAEIIQESMEIHQLICEAASTANKYFTYQLLTIISIAFLIIVFDAYYVLETLLGKSK
RESKFKTVEFVTFFSCQMILYLIAIISIVEGSNRAIKKSEKTGGIVHALLNKAKTADVKEKLQQFSMQLMHLKINFTA
AGLFNIDRTLYFTISGALTTYLIILLQFTSNSPPVTQNGCDPALAVSLNQTET

LcGr28bd

MKSFCYEFFKPKDSYSAERTLLAITFIMGLTPLRVRGSEGKRQMSISYLGFGITVLQGIFFVYCFIHSFLVDESIVGFF
FKTEISKIGDILQKFIGLSGMLILFGVSLRKSRDMIYMYHTIAVIDSRFLNLGVEFNYRYIMKFRHSKLIMVTIVCVG
YLASSLWMLFHNQIWPSFQAVVAFFVPHIFLLCVVVLNVSFVMRFWQHFDLLNKVLKNLCHQWDTRSIKTITH
KQRSLQCLDSFSMYTIVSKNPAEIIQESMEIHQLICEAASTANKYFTYQLLTIISIAFLIIVFDAYYVLETLLGKSKRESK
FKTVEFVTFFSCQMILYLIAIISIVEGSNRAIKKSEKTGGIVHALLNKAKTADVKEKLQQFSMQLMHLKINFTAAGLF
NIDRTLYFTISGALTTYLIILLQFTSNSPPVTQNGCDPALAVSLNQTET

LcGr28be

MCVTTIIPLSIRIFLHDLHKPRDVYACYRVLYLVALLTGVIPFQFTSQPNRLLKNTVFGYLNVFVRIVFYSFIFICSMSD
NEQSLLAHFYHTEVSRFTDSLQKFNGMFAILLVLIFGLGKRNALISLMQQYEVVELHFSRLGVQFQQKRCAWRIN
LTILIMFLANCSFIIYGHFVIFVHNNIYLSWIAIFSFYSPHTIISSVVVVFYTSLFKITHYFKAVNEVLKNLCHQWDTRS
IKTITHKQRSLQCLDSFSMYTIVSKNPAEIIQESMEIHQLICEAASTANKYFTYQLLTIISIAFLIIVFDAYYVLETLLGKS
KRESKFKTVEFVTFFSCQMILYLIAIISIVEGSNRAIKKSEKTGGIVHALLNKAKTADVKEKLQQFSMQLMHLKINFT
AAGLFNIDRTLYFTISGALTTYLIILLQFTSNSPPVTQNGCDPALAVSLNQTET

Sugar receptors

LcGr5a

MNGFSSACRGNTNVKCIFTLLALITKNIKPKCEQTCTCGMRYARDKDLLHNGSFHEAIGPILVMAQCFCLMPVR
GILSKSAKGLSFSWYSFRTFFCLLYMLTTILDTMLTTYKIVHNKLDVRNIEPFIFHFSILLASIGFLRLASKWPQLMR
RWQEVEEQLPPFNNWVEKEELAIRIKTVTFVLITLSLTEHLLSTISVIHFANYCPSDSDPIKSFFLTVVDQIFYIFDYS

PWLAWMGKIQNILLTFGWTYMDVFVMNVGIGLSSMFKRVQTHMENVQGLEMRESFWAKCRYQYTLICNLI
DEVDAAVSGIIMLSFANNLYFVCIQCLKSIKQVKMPSIAHAIYFYFSLMFLLARTLAVSLYLAEVNDRSRNPLTVIK
QIPPNCYYPEVERFVLEININKVAMTGMQYFDVTRKLVLTVAGTIVTYELVLIQFHEDQKPWHCGTD

LcGr61a
NTRKFFVLHSIKSFKHLDTFHRATRPCLLFAHIFGVMPLVNTWEYNPYRLKFNVPCWAMTTTVLFYIFGGWKT
MHFTEQMFKSGINARNIVGIVFFYMSFIISINFLNLARNWPQLIQYWTRIDLLFLMPPYKPPKWSLRKQLYTLLIG
FWITALVEHCLFYASGYYNFRMRRIHCHPDEEKYSFKDYIQLDIFTDIFIYFPYNIFVAVYAFFLSGTFTFLWNFLDF
FIMCISLGLATRFQQFNTRIEVLAGCYVPDAVWYKIRREHIILCEFMEKVNDQISTIVLLSSLNNMYFICNNLLNIF
TKLRYQINYVYFWFSLIFLLSRTICVFMFASKIHEASVLPLQTLYLVPTGCWTEEVQRFRAQILNEFIGLTGKRFYG
MTRKNLFGMMATIITYELMLLQLDNKNKENSLPELCT

LcGr64a
YNKTPVENFSVPDDSFSVIKPNKNYVVEENFNEVDDGEIKNSSEDTFHRAVSPILFLGQCFALMPVMGVLNPNP
KRLKFSYKSVQVIFTLFFLCSSSILTLTMLKFLVKVGINAKNFVGLVFFSCVQCSTLLFASLAPRWPNIMCYWSRVE
TIFTQKPYEMPKQPLASRVRISALLIFIGSAVEHALYLCSAVISYRRGIELCASVRNYTETVSFEDYVHRNYNYVFEIL
PYNMSVGVFILIVNGYCTFIWNYMDLFIMMVSKGIAYRFEQVTKRINNLKDKEIPETVFIEIREHYVKLCELLEFID
DNLSGIILLSCMNNLYFVCYQLLNIFNKLRWPINYIYFWYSLLYLIVRTAFVFLSAATINDESKGGLSVLRKVSSRT
WCVEVERLIFEMTTQTVALSGKKFYFLTRRLLFGMAGTIVTYELVLLQFDEPNRAKGLTALCG

LcGr64b
LFISQIFALLPVTNVYNKNLLKLKYSWISIPTCYAAIIMILNLSEFGFVIKYIFKTGVNFHTSGTISLFSVCLLEHVYLW
RLAIKWPNLMLQWRQVEQIFLKAPYKLYANYNMKLRIYFWYSLIMLCSLAEHMILLSNSFDKTQLEHKQCNLNT
SFWESLYARERPHLSGVLPCHYLILGILEWINLTLAYPRSFTDTFIIIVSIGLATRFRQLYLRIDSVKGKAMPSIFWLE
TREHFLVLKRLMRLISNELSPLILLALANNMYFICFQLFNSFNNIGVDSVAEIGFWFSLIFNIIRTILTLYLATLTNDYT
KKIVSCLRDIPSKSWCIETQRFSEQLSVDLTAFTGGGFFFMTRKLILAMASTIVTYELMVSDVINQGSIKQITDYCS
HYYDEPTDTLEQYS

LcGr64c
MLQVKVQSSDESESVKNTLHHALGPFLVLSRFFGIFPITGAWPTTDVEKVTFKWLTLPVLSTAVMMGFATLDV
CLTFRMMSEQGLKLTTTGPLSFSIECLSAFIVFLCLSRNWPNLIKSTRRLENIFSQHLYTCKESKKFSQRIRFLGWM
FLFCSVIEHSFYVSSGIYSNYLQIKQCNLTVNFWHNYYVRERLQIFSLVSYTVWLVPLLQWITISMTFVWNFIDIFL
ILTCRGLAIRFRQFDWCIKCHIKKNMPNDFWLKRRQDFLTLSDLFQQYDDKLSNLVLLSSAQNMYFMASHTFYI
FHLTRENFMMDVYFWFSFAFVAFRSFYMMLTASRIHDTTNEIIASMYEIPTAHWCLELKRLNEVIVSDLFAISGR
GFFFITRRLMLAMAGTLVIYELVLLDQVDSTDVTTPLCNQRKG

LcGr64f
DYTYTGSFQEAIRPVLILAQIFAIMPLAGVTSSSSYDLKFSWRFIRTWYSVLVILCFGFFMGVTIAYAFRGIFNFDS
VEGIIFYSSIFFIALTFFDMTRKWPMLMQEWQQVEQALPQQRTIMERSWLPHKIKMITLVATMCSLAEHLLSCI
SVIHYSNFCPITNDPIENFFRLSNEHIFKIFPYSNWWGWYGKISNFISTFTWNYMDIFVMIISIGLASKFQQLNEN
LMQYKNKQMPPAFWSENRILYRNLCSLCERMDDAISMITMVSFSNNLYFICVQLLRSLNKMPSIAHAAYFYFSL
FFLLGRTLAVSLYSSTIHDESRKPLLILRCVPKESWCLVVKRFAEEISNDLIALSGMKFFHLTRKLVLSVASTIVTYEL
VLIQFHEDTNLWDCENDRNQQFDYQNEMVKHLNGT

RNA Taste is Conserved in Dipterian Insects: Shinsuke Fujii et al.,

Supplementary Table 1

Query	Query ID	Length	Description	Total Score	Query cover	% Identity	Accession Length	Accession
**RNA/ribose Receptors**								
LcGr28a isoform X	XP_023298000.2	443	DmGr28a	536	99%	73.36	450	NP_523504.2
LcGr28ba	this study	450	DmGr28bb	502	92%	63.31	443	NP_995643.1
LcGr28bb	this study	447	DmGr28bb	676	93%	79.67	443	NP_995643.1
LcGr28bc	XP_046812333.1	454	DmGr28bc	639	94%	72.9	470	NP_995642.1
LcGr28bd	this study	438	DmGr28bd	582	94%	71.57	440	NP_995641.1
LcGr28be	this study	449	DmGr28be	499	91%	61.15	447	NP_995640.1
AaGr19aa	NP_001345039.1	444	DmGr28a	201	94%	32.64	450	NP_523504.2
AaGr19ab	EJY57898.1	444	DmGr28bb	195	93%	31.98	443	NP_995643.1
AaGr19ac	NP_001345038.1	449	DmGr28bb	199	91%	32.6	443	NP_995643.1
AgGr33	XP_319373.1	443	DmGr28bb	259	94%	35.71	443	NP_995643.1
**Larval/internal Fructose Receptors**								
LcGr43a	XP_046809331.1	450	DmGr43ac	442	100%	54.45	453	NP_001286158.1
AaGr34 isoform 2	NP_001345882.1	439	DmGr43aa	301	96%	41.19	427	NP_523650.2
AgGr25	XP_318100.4	432	DmGr43aa	297	98%	39.91	427	NP_523650.2
**CO2 Receptors**								
LcGr21a	XP_023299637.2	452	DmGr21ab	759	99%	82.63	447	NP_001259841.1
AaGr1	NP_001345045.1	460	DmGr21ab	639	93%	69.63	447	NP_001259841.1
AgGr22	XP_319142.1	467	DmGr21ab	627	90%	68.87	447	NP_001259841.1
**Bitter Receptors**								
LcGr66a	XP_023307046.2	529	DmGr66a	568	93%	59.1	527	NP_523971.3
AaGr14	NP_001345053.1	507	DmGr66a	287	89%	37.68	527	NP_523971.3
AgGr2	XP_307905.5	503	DmGr66a	300	96%	34.84	527	NP_523971.3
**Sugar Receptors**								
LcGr5a	this study	445	DmGr5a	419	99%	48.76	444	NP_511050.1
LcGr61a	this study	418	DmGr61ab	412	95%	49.62	436	NP_001261226.1
LcGr64a	this study	447	DmGr64a	562	100%	60.04	456	NP_728920.1
LcGr64b	this study	404	DmGr64b	469	97%	56.12	406	NP_728921.1
LcGr64c	this study	419	DmGr64cb	384	98%	44.93	419	NP_001246610.1
LcGr64f	this study	415	DmGr64f	451	93%	57.95	469	NP_728924.2
AaGr6	NP_001345147.1	441	DmGr64a	169	88%	30.77	456	NP_728920.1
AaGr7	NP_001345151.1	434	DmGr64a	276	93%	38.88	456	NP_728920.1
AaGr8	NP_001345159.1	377	DmGr64a	172	86%	31.5	456	NP_728920.1
AaGr9	NP_001345044.1	434	DmGr64f	216	98%	32.8	469	NP_728924.2
AaGr10	NP_001345051.1	455	DmGr64b	197	86%	29.43	406	NP_728921.1
AaGr5	NP_001345107.1	434	DmGr64ea	234	91%	30.42	451	NP_728923.2

AaGr4	NP_001345180.1	458	DmGr64f	353	86%	44.67	469	NP_728924.2
AgGr14	XP_316432.1	444	DmGr64a	231	95%	32.04	456	NP_728920.1
AgGr15	XP_307760.4	447	DmGr64f	328	88%	42.03	469	NP_728924.2
AgGr16	XP_307762.3	416	DmGr64ea	205	93%	31.89	451	NP_728923.2
AgGr17	XP_307763.1	410	DmGr61ab	205	98%	32.19	436	NP_001261226.1
AgGr18	XP_307764.3	476	DmGr64a	203	79%	31.93	456	NP_728920.1
AgGr20a	XP_307767.2	448	DmGr64a	192	87%	29.65	456	NP_728920.1
AgGr20b	XP_003436428.1	439	DmGr64a	190	87%	29.82	456	NP_728920.1
AgGr21	XP_307768.1	472	DmGr64a	278	84%	37.62	456	NP_728920.1
AgP3258	XP_307766.1	414	DmGr64a	165	95%	28.22	456	NP_7289A2:I5020.1
AgGr20a	XP_307767.2	448	DmGr64a	192	87%	29.65	456	NP_728920.1
AgGr20b	XP_003436428.1	439	DmGr64a	190	87%	29.82	456	NP_728920.1
AgGr21	XP_307768.1	472	DmGr64a	278	84%	37.62	456	NP_728920.1
AgP3258	XP_307766.1	414	DmGr64a	165	95%	28.22	456	NP_7289A2:I5020.1

The best matches of all identified homologs of the five groups (RNA/ribose Receptors, Internal/larval fructose Receptor, CO2 Receptor, bitter receptor and sugar Receptor) between *L. cuprina* and *D. melanogaster*, *Ae. aegypti* and *D. melanogaster*, and *A. gambiae* and *D. melanogaster* are shown. The pairs highlighted in colors (blue for *L. cuprina-D. melanogster*; orange for *Ae. aegypti- D. melanogster* and red for *A. gambiae- D. melanogaster*) identify the pairs of each group with the highest homology between the species and were used in Figure 3. Clustal Omega, the EMBL-EBI multiple sequence alignment tool, was used to determine all similarity parameters.

RNA Taste is Conserved in Dipterian Insects: Shinsuke Fujii et al.,

Supplementary Table 2

	Identity (%)	Similarity (%)	Gaps (%)	Scores
Gr28a				
Gr28b.a	29.6	49.1	13.3	564.5
Gr28b.b	34.8	54.6	11.6	672.5
Gr28b.c	30.9	52.0	15.3	667.0
Gr28b.d	33.9	51.4	12.6	629.5
Gr28b.e	29.5	50.5	17.3	529.0

Similarity of the *D. melanogaster* Gr28b proteins to Gr28a: Amino acid identity ranges between 29.5 and 34.8%, a range similar to the *Aedes* and *Anopheles* homologs to *Gr28a* and *Gr28bc*.
